# The effect of endoscopic renal and ureteral stone surgeries on renal blood flow in children: a prospective trial

**DOI:** 10.1007/s00240-024-01578-z

**Published:** 2024-06-07

**Authors:** Fevzi Batuhan Topbas, Cagri Akin Sekerci, Efe Soydemir, Ozge Yapici, Serkan Akbas, Selcuk Yucel, Tufan Tarcan, Yiloren Tanidir

**Affiliations:** 1 Department of Urology, School of Medicine, Marmara University, Istanbul, Turkey; 2 Department of Urology, Division of Pediatric Urology, School of Medicine, Marmara University, Istanbul, Turkey; 3 Department of Radiology, School of Medicine, Marmara University, Istanbul, Turkey; 4 Department of Urology, School of Medicine, Koç University, Istanbul, Turkey

**Keywords:** Urinary system stone disease, Children, Endourology, Doppler ultrasound, Blood perfusion

## Abstract

Aim: To assess the impact of endoscopic stone surgeries on renal perfusion and blood flow in children. Materials and methods: Children who underwent percutaneous nephrolithotomy (PCNL), retrograde intrarenal surgery (RIRS), ureterorenoscopy (URS), endoscopic combined intrarenal surgery (ECIRS) were included to the study. Renal Doppler ultrasonography (RDUS) was performed one day before the operation, and on the postoperative 1st day and 1st month. Peak systolic velocity (PSV) and end-diastolic velocity (EDV) were measured, and resistive index (RI) was calculated with the (PSV-EDV)/PSV formula. RDUS parameters were compared before and after surgery and between ipsilateral and contralateral kidneys. Results: A total of 45 children with a median age was 8 (2–17) years were included (15 (33.3%) girls, 30 (66.7%) boys). PCNL was performed in 13 children (28.9%), RIRS 11 (24.4%), URS 12 (26.7%), and ECIRS 9 (20%). There was no significant difference in renal and segmental PSV, EDV and RI values of operated kidney in the preoperative, postoperative periods. There was no significant difference between RDUS parameters of the ipsilateral and contralateral kidneys in preoperative or postoperative periods. PSV and EDV values were significantly higher in the 1st postoperative month in the group without preoperative DJ stent than in the group with DJ stent (*p* = 0,031, *p* = 0,041, respectively). However, RI values were similar. The mean RI were below the threshold value of 0.7 in each period. Conclusion: RDUS parameters didn’t show a significant difference in children. Endoscopic surgeries can be safely performed in pediatric stone disease.

**Supplementary Information:**

The online version contains supplementary material available at 10.1007/s00240-024-01578-z.

## Introduction

In recent years, with the development of biomedical technologies, the widespread use of endoscopic treatments in pediatric stone disease has emerged. While these treatments offer significant contributions to stone management, it is acknowledged that certain challenges are still encountered during their application. One of these challenges is the elevation of intrarenal pressure resulting from the use of irrigation fluid in endoscopic surgeries, such as ureteroscopy (URS), retrograde intrarenal surgery (RIRS), and percutaneous nephrolithotomy (PCNL). In endourological interventions. Normal intrarenal pressure is around an average of 10 mmHg [1]. It has been demonstrated that pyelorenal reflux begins at pressures of 30–45 mmHg [2, 3]. In these surgeries, instruments of various diameters and qualities can be used, and these differences can impact the pressure within the collecting system.

In a study conducted in 2018, it was indicated that in adult patients undergoing RIRS, a difference of more than 1.5 Fr between the inner diameter of the ureteral access sheath (UAS) and the diameter of the ureteroscope is safe for renal blood flow [4]. Another study published in 2021 revealed that in patients undergoing RIRS, the peak systolic velocity (PSV) and resistive index (RI) values were significantly higher in the postoperative period compared to the preoperative period [5]. In addition, in adult patients undergoing PCNL, it has been found that the RI measured from the lower pole in renal Doppler ultrasound (RDUS) performed in the early postoperative period is significantly higher in patients with nephrostomy compared to those without nephrostomy [6]. Furthermore, it has been reported that the resistive index (RI) measured in the arcuate arteries of the accessed calyx is similar to those in other calyces, but significantly higher than RIs of the three calyces in the contralateral kidney [7]. . However, in another study, no significant difference was observed between RIs in the accessed calyx, other calyces, and the calyces of the contralateral kidney [8]. . In addition, it was observed that the RI decreased significantly on the 7th postoperative day in patients with a kidney with partial obstruction due to ureteropelvic junction stone and an RI ≥ 0.7 [9]. To our knowledge, there are no studies in the literature on this subject in pediatric age group. The aim of this study is to evaluate the effects of endoscopic stone surgeries (PCNL, RIRS, URS, ESWL) on renal perfusion and blood flow in children using RDUS.

## Materials and methods

The children who underwent endoscopic surgeries (URS, PCNL, RIRS, and endoscopic combined intrarenal surgery - ECIRS) due to urinary stone disease at our clinic between March 2021 and December 2022 were included in this study. This prospective study was designed without any randomization. Ethical approval was obtained from the local ethics committee before the study (Ethical number: 09.2021.487). Children with hypertension, congenital heart disease or those using antihypertensive medications, individuals with a history of vasculitis, patients over 18 years and below 2 years were excluded from the study. Children under 2 years of age were excluded due to their agitation and noncompliance during ultrasound. PSV, end-diastolic velocity (EDS) of the segmental and renal arteries of both kidneys were measured by RDUS on the day before surgery and on the 1st day and 1st month of surgery. RI was calculated with the (PSV-EDV)/PSV formula. RDUS parameters of the ipsilateral and contralateral kidneys in the preoperative, early postoperative period, and 1st postoperative month were compared. Additionally, subgroup analyzes were performed according to preoperative, peroperative and postoperative parameters and surgery types.

### Surgical techniques and instruments

A Richard Wolf brand Ultra-Thin semirigid ureterorenoscope with a 5-degree angle, a distal tip diameter of 4.5 Fr, a body diameter of 6.5 Fr, and a working channel of 3.3 Fr was used for URS. In patients undergoing RIRS, a flexible ureterorenoscope, Karl Storz Flex X2S model, with a distal tip diameter of 6.6 Fr, a shaft diameter of 7.5 Fr, and a working channel diameter of 3.6 Fr, capable of 270 degrees flexion and deflection, was employed. In PCNL, two types of instruments were used: the Ultra-Thin ureterorenoscope mentioned above and an Olympus 15 Fr mini nephroscope with a 7-degree angled and 6 Fr working channel. The Ho: YAG and Thulium Fiber Laser (TFL) were utilized for laser lithotripsy. The Ho: YAG laser, operated with a Quanta Litho model with a frequency of 3–25 Hz, energy ranging from 0.2 to 4 J, and a 30 W laser system emitting a 2.1 μm wavelength. The TFL, on the other hand, was employed with a Quanta Cyber Ho model, capable of reaching a frequency of 60 Hz and energy up to 5 J, using a 60 W laser system emitting a 2.1 μm wavelength. Lithotripsy was performed with 272 and 365 μm laser fibers.

PCNL was performed in the Galdakao-modified supine Valdivia position. Endoscopic entry through the urethra was used to pass a guide wire from the ureteric orifice of the side to be operated on to the renal pelvis. Subsequently, a 5 or 6 Fr ureteral catheter was advanced proximally into the ureter, and contrast material (iohexol) diluted with 50% physiological saline was injected. The collecting system was visualized under fluoroscopy, and access to the collecting system was achieved using this image. In some cases, this was supplemented by ultrasonographic images obtained with a 3.5 MHz ultrasound probe. The targeted calyx was entered with an 18-gauge needle, and after ensuring access to the collecting system, a guide wire was sent through the needle. Dilation was performed using telescopic metal dilators. Following dilation, a trocar or Amplatz sheath compatible with the anatomy of the stone and kidney was placed, and lithotripsy was initiated using an instrument advanced through it.

RIRS and URS were performed in the lithotomy position. In patients undergoing RIRS, similar to PCNL, lithotripsy was performed either directly through a flexible ureterorenoscope sent over the guide wire or through a flexible ureterorenoscope advanced through the ureteropelvic junction. In patients undergoing URS, lithotripsy was performed either directly over or alongside the guide wire using a semi-rigid ureterorenoscope, reaching the level of the stone.

### Renal doppler ultrasound

RDUS was performed in the radiology department of our hospital using a Samsung RS 85 model device with a C1-5 convex probe by an experienced radiologist. Prior to the examination, renal arteries were assessed for possible stenosis or anatomical anomalies. Renal artery velocity measurements were taken from the point before branching. This approach helped to mitigate the effects of surrounding structures and prevent inaccurate results. PSV and EDV values of the arcuate and segmental arteries were measured using sagittal images, which provided the most suitable angle and reliable measurements. RI values were obtained using the formula (PSV-EDV)/PSV. PSV shows the greatest flow velocity in an artery throughout the systole. It provides direct assesment of the arterial supply. Low PSV indicates deterioration in arterial flow. EDV and RI indirectly reflects arterial compliance and resistance [10, 11]. 

### Statistical analyses

IBM SPSS (Statistical Package for the Social Sciences) version 23.0 was utilized for statistical analyses. The normal distribution of the data was assessed through the Shapiro-Wilk test. Since the data did not exhibit a normal distribution, non-parametric methods were employed. Nominal data were presented in tables as median, minimum, and maximum values. The comparison of RDUS values in the preoperative, postoperative periods of the effected kidneys was conducted using Friedman test. The comparison of RDUS values between the affected kidneys and contralateral side was performed using Mann-Whitney U test. Significant p value was considered as 0.05.

## Results

A total of 45 children were included in the study. 15 (33.3%) of the children were girls, 30 (66.7%) were boys, and the median age was 8 (2–17) years. The number of children with preoperative double J stents was 21 (46.7%), and the number of children with preoperative hydronephrosis was 10 (22.2%). Nine patients (20%) had a history of urinary tract infections, and 14 patients (31.1%) had a previous intervention for urinary system stones. Among these, 6 had URS, 2 PCNL, 2 Shock Wave Lithotripsy (SWL), 1 ECIRS. Additionally, three patients had a history of multiple stone surgeries (1 patient had URS, RIRC, and PCNL; 1 patient had RIRS and PCNL; 1 patient had RIRS and SWL). Height, weight, stone size, stone volume, stone location, number of stones, stone side, Hounsfield Units (HU) and Guy stone score of children are given in Supplementary Tables 1–2. Thirteen patients (28.8%) underwent PCNL, 11 (24.4%) RIRS, 12 (26.7%) URS and, 9 (20%) ECIRS. Surgical side, accessed calyx, imaging method, Amplatz sheath size, energy source **types** are given in Supplementary Table 3.

There was no statistically significant difference observed in the preoperative, early postoperative 1st -day, and postoperative 1st -month PSV, EDV, and RI values of the renal and segmental artery in the affected kidney (Table 1). The preoperative, postoperative 1st -day, and 1st -month PSV, EDV, and RI values were similar between the ipsilateral and contralateral kidneys (Table 2). When examined according to surgical types, no differences were observed in the PSV, EDV, and RI values of the affected kidney (Table 3). When compared based on the preoperative stent presence, a difference was observed only in the segmental PSV and EDV values during the first postoperative month (*p* = 0.031, *p* = 0.041, respectively) (Table 4). No difference was detected between RDUS values according to the presence of hydronephrosis (Supplementary Table 4). When compared in terms of stone burden and previous interventions for urinary system stones, all parameters except Postoperative 1st month Renal RI were similar between the two groups (*p* = 0.026, *p* = 0.048, respectively) (Supplementary Tables 5–6).

**Table 1:** Comparison of preoperative, postoperative first day and month RDUS parameters of the affected kidney (*n* = 45)

	Preoperative		Postoperative 1. day		Postoperative 1. month		*P* value
	Median	Min.-Max.	Median	Min.-Max.	Median	Min.-Max.	
**Segmental PSV**	30.41	16.05–79.8	30	18-94.47	33.46	16.4-98.89	0.664
**Segmental EDV**	11.2	5.07–39.16	11.66	5.46–39.6	11.95	5.9-40.42	0.761
**Segmental RI**	0.62	0.38–0.75	0.61	0.46–0.76	0.63	0.43–0.78	0.283
**Renal PSV**	88.9	32.4-159.6	72	34.6-199.81	79.75	34.9-194.06	0.600
**Renal EDV**	30.84	8-67.9	27	11.4–64.5	25.69	10.3-73.14	0.978
**Renal RI**	0.65	0.48–0.81	0.65	0.49–0.86	0.66	0.47–0.88	0.508

PSV: Peak systolic velocity, EDV: End-diastolic velocity, RI: Resistive index

**Table 2:** Comparison of preoperative, postoperative first day and month RDUS parameters of the ipsilateral and contralateral kidney (*n* = 45)

	Preoperative			Postoperative 1. day			Postoperative 1. month		
	Ipsilateral Median (Min.-Max.)	Contralateral Median (Min.-Max.)	*P* value	Ipsilateral Median (Min.-Max.)	Contralateral Median (Min.-Max.)	*P* value	İpsilateral Median (Min.-Max.)	Contralateral Median (Min.-Max.)	*P* value
**Segmental PSV**	30.41 (16.05–79.8)	34.9 (21–72)	0.069	30 (18-94.47)	30.05 (21-119.53)	0.741	33.46 (16.4–98.8)	30 (19.3-92.01)	0.628
**Segmental EDV**	11.2 (5.07–39.16)	12.8 (8.16–30.5)	0.120	11.66 (5.46–39.6)	11.8 (6.93–37.84)	0.640	11.95 (5.9-40.42)	11.9 (5.97–30.96)	0.669
**Segmental RI**	0.62 (0.38–0.75)	0.63 (0.49–0.7)	0.728	0.61 (0.46–0.76)	0.6 (0.5–0.74)	0.765	0.63 (0.43–0.78)	0.64 (0.38–0.75)	0.398
**Renal PSV**	88.9 (32.4-159.6)	86.18 (35.2-192.61)	0.881	72 (34.6-199.81)	81.4 (37.6-172.53)	0.417	79.75 (34.9–194)	98.41 (38-207.66)	0.264
**Renal EDV**	30.84 (8-67.9)	29.3 (7.1–65.7)	0.949	27 (11.4–64.5)	28.72 (13.2-60.84)	0.153	25.69 (10.3–73.1)	32.51 (10.7-85.15)	0.152
**Renal RI**	0.65 (0.48–0.81)	0.66 (0.48–0.79)	0.965	0.65 (0.49–0.86)	0.62 (0.47–0.75)	0.314	0.66 (0.47–0.88)	0.67 (0.45–0.82)	0.648

PSV: Peak systolic velocity, EDV: End-diastolic velocity, RI: Resistive index

**Table 3:** Comparison of preoperative, postoperative first day and month RDUS parameters of the affected kidney according to types of surgeries

		Preoperative		Postoperative 1. day		Postoperative 1. month		*P* value
		Median	Min.-Max.	Median	Min.-Max.	Median	Min.-Max.	
**PCNL** **(** *n* **= 13)**	Segmental PSV	37.12	20-74.7	39.09	23.4-94.47	29.4	16.4-65.17	0.662
	Segmental EDV	16.1	7.68–25.2	15.44	9.4-32.74	12.03	6.4-26.07	0.397
	Segmental RI	0.6	0.5–0.74	0.6	0.46–0.68	0.61	0.48–0.69	0.657
	Renal PSV	92.8	36-218.1	72	34.6-177.07	75.01	34.9-194.06	0.943
	Renal EDV	32.6	8-42.5	25.34	14-48.13	26.1	10.7-64.44	0.943
	Renal RI	0.64	0.54–0.77	0.64	0.51–0.73	0.66	0.59–0.71	0.156
**RIRS** **(** *n* **= 11)**	Segmental PSV	24.19	16.05–60.5	24.3	18-60.61	26.61	18.8-55.76	0.850
	Segmental EDV	8.98	5.07–18.8	10.14	5.46–15.95	10.14	5.9-22.45	0.739
	Segmental RI	0.65	0.54–0.74	0.64	0.5–0.74	0.64	0.53–0.75	0.497
	Renal PSV	93.7	59.87-117.27	80.4	36.9-136.31	95.38	40.2-167.99	0.534
	Renal EDV	26.58	11.48–43.7	27	11.56–43.94	25.15	10.3-59.33	0.534
	Renal RI	0.69	0.57–0.81	0.67	0.54–0.86	0.68	0.57–0.76	0.395
**URS (n: 12)**	Segmental PSV	28.98	20.09–79.8	30.82	23.9–79.1	38.65	18.8-98.89	0.264
	Segmental EDV	10.91	6.11–39.16	11.13	5.97–39.6	13.64	7.9-40.42	0.205
	Segmental RI	0.63	0.38–0.75	0.61	0.5–0.76	0.61	0.43–0.78	0.262
	Renal PSV	85.98	32.4-159.6	79.17	36.1-199.81	102.71	37.2-169.3	0.717
	Renal EDV	25.195	8.3–67.9	27.62	11.4–64.5	31.95	12.9-73.14	0.920
	Segmental PSV	28.98	20.09–79.8	30.82	23.9–79.1	38.65	18.8-98.89	0.264
**ECIRS** **(n: 9)**	Segmental PSV	34.27	25.34–40.88	28.49	24-47.4	33.56	22-56.9	0.459
	Segmental EDV	13.12	6.91–17.2	11.66	7.77–15.4	12.9	9.9–19.5	0.641
	Segmental RI	0.61	0.53–0.74	0.61	0.52–0.72	0.61	0.55–0.68	0.549
	Renal PSV	81.94	48.1-102.7	64	37.6-95.32	67.11	35.9-175.23	0.368
	Renal EDV	31.26	14-40.1	27.4	15–30	24.5	12.1-57.93	0.139
	Renal RI	0.64	0.55–0.71	0.64	0.52–0.75	0.67	0.58–0.71	0.074

PCNL: percutaneous nephrolithotomy, RIRS: retrograde intrarenal surgery, URS: ureterorenoscopy, ECIRS: endoscopic combined intrarenal surgery, PSV: Peak systolic velocity, EDV: End-diastolic velocity, RI: Resistive index

**Table 4:** Comparison of preoperative, postoperative first day and month RDUS parameters of the affected kidney according to presence of double J stent

	DJ stent was not placed (*n*: 21)		DJ stent placed (*n*: 24)		*P* value
	Median	Min.-Max.	Median	Min.-Max.	
**Preop Segmental PSV**	36.9	20-79.8	30.205	16.05–74.7	0.345
**Preop Segmental EDV**	12.67	6.11–39.16	11.02	5.07–25.2	0.176
**Preop Segmental RI**	0.6	0.38–0.75	0.64	0.5–0.74	0.085
**Preop Renal PSV**	86.1	34-159.6	90.905	32.4-137.71	0.649
**Preop Renal EDV**	30.84	13.1–67.9	29.93	8-48.31	0.955
**Preop Renal RI**	0.64	0.48–0.78	0.66	0.54–0.81	0.129
**Postop 1st day Segmental PSV**	33.24	23.4–79.1	26.905	18-94.47	0.116
**Postop 1st day Segmental EDV**	12.4	5.97–39.6	11.63	5.46–32.74	0.211
**Postop 1st day Segmental RI**	0.59	0.5–0.76	0.63	0.46–0.74	0.214
**Postop 1st day Renal PSV**	78.2	34.6-125.6	67.23	36.9-199.81	0.794
**Postop 1st day Renal EDV**	26.6	11.4–64.5	27.05	11.56–63.98	0.776
**Postop 1st day Renal RI**	0.65	0.49–0.79	0.655	0.52–0.86	0.991
**Postop 1st month Segmental PSV**	37.01	18.6-98.89	29.05	16.4–56.9	**0.031**
**Postop 1st month Segmental EDV**	14.1	7.5-40.42	10.995	5.9-22.45	**0.041**
**Postop 1st month Segmental RI**	0.63	0.43–0.78	0.63	0.53–0.75	0.758
**Postop 1st month Renal PSV**	86.84	34.9-194.06	77.465	35.9-175.23	0.964
**Postop 1st month Renal EDV**	25.33	10.7-73.14	26.015	10.3-59.33	0.733
**Postop 1st month Renal RI**	0.66	0.47–0.88	0.665	0.57–0.76	0.882

PSV: Peak systolic velocity, EDV: End-diastolic velocity, RI: Resistive index, DJ: Double J. Postop: Postoperative

In postoperative X-rays, no residual stone fragments were observed in 38 patients (84.5%), while a single fragment was seen in 5 patients (11.1%), and multiple fragments were detected in 2 patients (4.4%). The number of patients with complications was 13 (28.9%), with one (7.6%) classified as Clavien-Dindo (CD) grade 1 and twelve (92.3%) as CD grade 2. No complications of CD grade 3 or higher were observed. Penile edema was noted as a CD grade 1 complication, while CD grade 2 complications included fever, positive blood culture, febrile convulsion, elevation in acute-phase reactants, and hemoglobin drop requiring erythrocyte sedimentation replacement. The mean length of hospital stay for patients was 3.42 ± 3.15 days.

## Discussion

In the present study, no significant difference was observed in PSV, EDV, and RI values in renal and segmental arteries in the RDUS examination performed before and after pediatric endoscopic stone surgeries. We believe that these findings are important indicators of the reliability of these increasingly used minimally invasive surgeries in children. The inevitability of the future widespread adoption of less complication-prone minimally invasive methods is anticipated, and maintaining low intrarenal pressure in these surgeries is particularly crucial for preventing infectious complications [12].

Platt et al. determined the threshold value for renal RI as 0.7 in their study involving adults, indicating an increase in renal vascular resistance when it exceeds 0.7 [13]. Bude et al., on the other hand, reported age-related variations in RI in healthy children. They found that renal RI in the first year of life was above the adult upper limit of 0.7 and decreased below this value with age, with the rate of RI measurements exceeding 0.7 after the age of 4 being 2% [14]. In another study, the renal RI was found to be 0.68 ± 0.06 in children under 1 year old, 0.66 ± 0.06 in the 1–9 age group, and 0.58 ± 0.05 in the 10–18 age group [15]. Cvitkovic-Kuzmic et al., in their study on renal RIs, found an average of 0.705 ± 0.018 in those under 6 years old, 0.605 ± 0.029 in the 6–16 age group, and 0.604 ± 0.035 in adults [16]. In another study, the mean renal RI was found to be 0.706 ± 0.066 in the 0–1 age group, 0.659 ± 0.05 in the 1–3 age group, 0.638 ± 0.042 in the 3–6 age group, and 0.608 ± 0.039 in the 6–16 age group, with no RI value exceeding 0.7 observed in patients aged 6 and above [17]. In the present study, the youngest patient was 2 years old, and renal RI values ranged between 0.62 and 0.67, similar to these studies.

A study evaluating 46 adult patients who underwent RIRS investigated the effects of combinations of UAS and flexible ureterorenoscopes on RDUS parameters. In this study, which RDUS was performed 2 days preoperatively and 24 h postoperatively, among the patients using a 9.5/11.5 Fr UAS and Karl Storz Flex X2 model ureterorenoscope, the RI value in the arcuate artery increased from 0.59 in the preoperative measurement to 0.62 in the postoperative measurement (*p* = 0.016). It has been reported that to preserve renal perfusion, there should be a 1.5 Fr difference between the inner diameter of the UAS and the diameter of the ureteroscope [4]. In our study, among the 11 patients who underwent RIRS, UAS was not used in 9 cases, while the procedure was performed with this combination in the other 2 patients. In a study by Rehman et al., on cadaveric kidneys, when ureteroscopy was performed using an UAS, intrapelvic pressure did not exceed 30 cmH2O, whereas without UAS, pressures exceeding 50 cmH2O were measured. Furthermore, the group undergoing RIRS without UAS showed a pressure increase ranging from 35 to 80% [18]. In our study, the limited use of an UAS prevented the evaluation of its impact on RDUS parameters, and therefore, an analysis predicting intrarenal pressure was not feasible.

In a study evaluating RDUS parameters of adult patients undergoing RIRS, PSV and RI values in the arcuate artery on the side of the operation increased from 48.19 to 53.55 and from 0.52 to 0.57 postoperatively, compared to preoperative values (*p* = 0.032 and *p* = 0.012, respectively). This elevation was thought to be associated with vasoconstriction in the minor vessels of the kidney. The EDV values in the arcuate artery remained similar to preoperative levels [5]. In our study, no significant changes were observed in the segmental or renal artery in patients undergoing RIRS. However, the mentioned study was conducted on 56 patients, while our study evaluated 11 patients undergoing RIRS. Vasoconstriction, aimed at reducing blood flow to the region, is the first event to occur to ensure hemostasis due to damage to the endothelial tissue [19]. In these surgeries, it is expected that the increased intraluminal pressure would affect the arcuate artery before the renal artery, and the postoperative increase in arcuate PSV can be explained in this way. In children, the vascular walls are thinner and more flexible compared to adults, resulting in shorter and milder vasoconstriction [20]. It has been considered that another reason for the absence of an increase in PSV and RI values on the operation side in children, as observed in adults, could be attributed to these differences.

In a study by Akın et al. comparing standard and tubeless PCNL in adults, the postoperative RI value in the lower calyx between in patients who underwent tubeless PCNL was found to be 0.666 ± 0.05, which was statistically significantly lower than the value of 0.698 ± 0.049 observed in patients undergoing standard PCNL (*p* = 0.013). However, no statistically significant difference in RI values was observed between standard and tubeless PCNL at postoperative 6th months [6]. In our study, all patients underwent tubeless PCNL, and therefore, a comparison with standard PCNL was not possible. In patients undergoing tubeless PCNL, similar preoperative, early postoperative, and postoperative 1st -month renal and segmental RI results were observed. This finding, consistent with the lack of changes in vascular resistance (RI) in long-term follow-up in the study conducted in adult patients, is also evident in our study, supporting the notion that vascular resistance remains stable over time.

In a study comparing preoperative and postoperative renal RI values in kidneys with ureteropelvic junction stones, in the group with preoperative RI values below 0.7, there was no significant decrease in RI values at postoperative 1st month, while in patients with RI values above 0.7, the values decreased from 0.73 to 0.61 at postoperative 1st month (*p* = 0.001) [9]. Obstruction in the upper urinary system is a pathology that, like endourological interventions, increases intraluminal pressure. There are studies indicating that obstruction raises renal RI [21]. In our study, the postoperative 1st month renal RI values in patients with preoperative hydronephrosis were statistically significantly *higher* compared to those without hydronephrosis. In this regard, our study reached similar results to other studies in the literature.

When we compared the patients with and without preoperative DJ stent, it was observed that PSV and EDV values were significantly higher in the 1st postoperative month in the group without DJ stent than in the group with DJ stent, but RI did not change. In patients who have undergone preoperative stent placement, the presence of the stent is expected to reduce intrarenal pressure by causing passive ureteral dilatation. The higher PSV and EDV values indicating vascular resistance and arterial perfusion in patients without stent can be explained in this way but the main indicator of vascular resistance is RI, and an increase in PSV and EDV values alone has no clinical significance [22, 23].

The study has some limitations. The most significant limitation is the limited number of patients. As it was not possible to measure intrarenal pressure during the operation, the pressure dynamics of the kidney could not be evaluated, and comparisons with RDUS parameters could not be made. Another parameter indicating vascular resistance, the pulsatility index, was not assessed in our study. RI measurements in our study were performed from the lower calyx, and a study evaluating the other calyces would be beneficial. The wide age range of the children is also a limitation. Although this was a prospective study, randomization was not applied in terms of treatment selection. The type of surgery was planned in accordance with the European Association of Urology guidelines.

## Conclusion

In some studies, conducted with adults, differences were observed in RDUS parameters between the operated kidney and the contralateral kidney following endourological interventions. However, present study concluded that endourological surgeries do not cause impairment in renal perfusion in children. We believe that endourological interventions, which currently constitute almost the entire surgical treatment for stone disease, can be safely applied in children as well, similar to adults. Nevertheless, we think that further studies with larger patient groups and more detailed ultrasonographic examinations should be designed.

## Electronic supplementary material

Below is the link to the electronic supplementary material.

Supplementary Material 1: table 1 The age, weight, height, Hounsfield Unit values and stone dimensions.

Supplementary Material 2: table 2 Distribution of stone-related parameters.

Supplementary Material 3: table 3 Distribution of peroperative parameters.

Supplementary Material 4: Table 4. Comparison of preoperative, postoperative first day and month RDUS parameters of the affected kidney according to presence of hydronephrosis.

Supplementary Material 5: table 5 Comparison of preoperative, postoperative first day and month RDUS parameters of the affected kidney according to stone burden.

Supplementary Material 6: table 6 Comparison of preoperative, postoperative first day and month RDUS parameters of the affected kidney according to history of previous intervention for urinary system stones

## Data Availability

No datasets were generated or analysed during the current study.
