# Supplementary material for: The effect of endoscopic renal and ureteral stone surgeries on renal blood flow in children: a prospective trial
Source: Urolithiasis. 2024 Jun 7;52(1):84. doi: 10.1007/s00240-024-01578-z (PMC11161530; doi:10.1007/s00240-024-01578-z)
Supplement: Supplementary file 1 — Supplementary Material 1: table 1 The age, weight, height, Hounsfield Unit values and stone dimensions. [file 240_2024_1578_MOESM1_ESM.docx]

**Supplementary Table 1.** The age, weight, height, Hounsfield Unit values and stone dimensions

|  | **Median (Min.-Max.)** |
| --- | --- |
| **Age** | 8 (2-17) |
| **Weight (kg)** | 27 (9-100) |
| **Height (cm)** | 120 (80-180) |
| **Preop DJ duration (days)** | 65 (8-165) |
| **Stone volume (mm^3^)** | 292 (14-2400) |
| **Stone surface area (mm^2^)** | 251 (30-1860) |
| **Maximum Hounsfield Unit** | 1127 (268-2812) |
| **Mean Hounsfield Unit** | 776 (180-1705) |
| **Stone anterior-posterior diameter (mm)** | 6.8 (3.1-22) |
| **Stone mediolateral diameter (mm)** | 8.3 (2.6-34) |
| **Stone superior inferior diameter (mm)** | 8.6 (3.6-28.6) |
| **Stone maximum diameter (mm)** | 9.4 (3.6-28.6) |
| **Stone-skin distance (mm)** | 54.5 (37-105) |

*DJ: Double J, HU: Hounsfield Unit*
