# Supplementary material for: The effect of endoscopic renal and ureteral stone surgeries on renal blood flow in children: a prospective trial
Source: Urolithiasis. 2024 Jun 7;52(1):84. doi: 10.1007/s00240-024-01578-z (PMC11161530; doi:10.1007/s00240-024-01578-z)
Supplement: Supplementary file 2 — Supplementary Material 2: table 2 Distribution of stone-related parameters. [file 240_2024_1578_MOESM2_ESM.docx]

**Supplementary Table 2.** Distribution of stone-related parameters

|  |  | **n** | **%** |
| --- | --- | --- | --- |
| **Stone side** | Left | 21 | 46.7 |
|  | Right | 21 | 46.7 |
|  | Bilateral | 3 | 6.7 |
| **Stone opacity** | Radiolucent | 8 | 17.8 |
|  | Semiopaque | 7 | 15.6 |
|  | Opaque | 30 | 66.7 |
| **Stone type** | One simple | 24 | 53.3 |
|  | Multiple simple | 15 | 33.3 |
|  | Semistaghorn | 3 | 6.7 |
|  | Semistaghorn + simple | 3 | 6.7 |
| **Stone localization** | Renal pelvis | 8 | 17.8 |
|  | Lower pole | 8 | 17.8 |
|  | Middle calyx | 1 | 2.2 |
|  | Multiple calices | 7 | 15.6 |
|  | UPJ | 2 | 4.4 |
|  | Proximal üreter | 1 | 2.2 |
|  | Mid ureter | 1 | 2.2 |
|  | Distal ureter | 9 | 20 |
|  | Both kidney and ureter | 8 | 17.8 |
| **Number of stones** | One | 24 | 53.3 |
|  | Multiple | 21 | 46.7 |
| **Guy’s stone score** | 1 | 11 | 35.5 |
|  | 2 | 14 | 45.2 |
|  | 3 | 5 | 16.1 |
|  | 4 | 1 | 3.2 |

*UPJ: Ureteropelvic junction*
