# Supplementary material for: The effect of endoscopic renal and ureteral stone surgeries on renal blood flow in children: a prospective trial
Source: Urolithiasis. 2024 Jun 7;52(1):84. doi: 10.1007/s00240-024-01578-z (PMC11161530; doi:10.1007/s00240-024-01578-z)
Supplement: Supplementary file 3 — Supplementary Material 3: table 3 Distribution of peroperative parameters. [file 240_2024_1578_MOESM3_ESM.docx]

**Supplementary Table 3.** Distribution of peroperative parameters

|  |  | **n** | **%** |
| --- | --- | --- | --- |
| **Side of PCNL** | Left | 4 | 30.1 |
|  | Right | 9 | 69.9 |
| **Side of RIRS** | Left | 8 | 72.7 |
|  | Right | 3 | 27.3 |
| **Side of URS** | Left | 4 | 33.3 |
|  | Right | 8 | 66.7 |
| **Side of ECIRS** | Left | 6 | 66.7 |
|  | Right | 3 | 33.3 |
| **Operation type** | URS | 11 | 24.4 |
|  | RIRC | 12 | 26.7 |
|  | PNL | 13 | 28.9 |
|  | ECIRS | 9 | 20 |
| **Access calyx** | Middle | 6 | 27.3 |
|  | Lower | 14 | 63.6 |
|  | Multiple (upper and middle) | 2 | 10.1 |
| **Imaging used for access** | Fluoroscopy | 9 | 40.9 |
|  | Fluoroscopy and USG | 13 | 59.1 |
| **Amplatz size** | 16 Fr | 10 | 45.5 |
|  | 20 Fr | 3 | 13.6 |
|  | 12 Fr | 3 | 13.6 |
|  | 3.5 mm trocar | 5 | 22.7 |
|  | 3 mm trocar | 1 | 4.6 |
| **Energy source** | TFL | 25 | 55.6 |
|  | Ho: YAG | 18 | 40 |
|  | Lithotripsy was not performed | 2 | 4.4 |
| **Instrument used in PNL** | Mini-nephroscope | 8 | 36.4 |
|  | Ultrathin URS | 14 | 63.6 |

*PCNL: Percutaneous nephrolithotomy, RIRS: Retrograde intrarenal surgery, URS: Ureterorenoscopy, ECIRS: Endoscopic combined intrarenal surgery, USG: Ultrasonography, TFL: Thulium fiber laser, Ho: YAG: Holmium Yttrium Aluminum Garnet*
