# Supplementary material for: The effect of endoscopic renal and ureteral stone surgeries on renal blood flow in children: a prospective trial
Source: Urolithiasis. 2024 Jun 7;52(1):84. doi: 10.1007/s00240-024-01578-z (PMC11161530; doi:10.1007/s00240-024-01578-z)
Supplement: Supplementary file 4 — Supplementary Material 4: Table 4. Comparison of preoperative, postoperative first day and month RDUS parameters of the affected kidney according to presence of hydronephrosis. [file 240_2024_1578_MOESM4_ESM.docx]

**Supplementary Table 4**. Comparison of preoperative, postoperative first day and month RDUS parameters of the affected kidney according to presence of hydronephrosis

|  | **Not hydronephrotic**  **(n: 35)** | | **Hydronephrotic**  **(n: 10)** | | **P value** |
| --- | --- | --- | --- | --- | --- |
|  | Median | Min.-Max. | Median | Min.-Max. |  |
| **Preop Segmental PSV** | 34.03 | 16.05-79.8 | 27.3 | 18-74.7 | 0.375 |
| **Preop Segmental EDV** | 12 | 5.07-25.3 | 10.765 | 7.2-39.160.62 | 0.838 |
| **Preop Segmental RI** | 0.62 | 0.5-0.75 | 0.61 | 0.38-0.66 | 0.325 |
| **Preop Renal PSV** | 89.8 | 32.4-159.6 | 76.675 | 37.7-128.1 | 0.633 |
| **Preop Renal EDV** | 30.84 | 8-67.9 | 29.145 | 13.1-44.71 | 0.733 |
| **Preop Renal RI** | 0.65 | 0.54-0.81 | 0.64 | 0.48-0.72 | 0.324 |
| **Postop 1^st^day Segmental PSV** | 30.44 | 19.69-79.1 | 27.46 | 18-94.47 | 0.806 |
| **Postop 1^st^ day Segmental EDV** | 12.1 | 5.46-39.6 | 11.135 | 7.2-32.74 | 0.712 |
| **Postop 1^st^ day Segmental RI** | 0.61 | 0.46-0.76 | 0.6 | 0.51-0.71 | 0.935 |
| **Postop 1^st^ day Renal PSV** | 72 | 34.6-199.81 | 70.4 | 36.1-177.07 | 0.692 |
| **Postop 1^st^day Renal EDV** | 27.1 | 11.56-64.5 | 24.11 | 11.4-48.13 | 0.692 |
| **Postop 1^st^ day Renal RI** | 0.65 | 0.49-0.86 | 0.66 | 0.57-0.73 | 0.46 |
| **Postop 1^st^month Segmental PSV** | 33.46 | 16.4-98.89 | 33.06 | 22-56.9 | 0.935 |
| **Postop 1^st^month Segmental EDV** | 12.29 | 5.9-40.42 | 11.21 | 9.32-19.5 | 0.604 |
| **Postop 1^st^month Segmental RI** | 0.61 | 0.48-0.78 | 0.66 | 0.43-0.7 | 0.126 |
| **Postop 1^st^month Renal PSV** | 86.84 | 34.9-194.06 | 75.095 | 35.9-127.02 | 0.353 |
| **Postop 1^st^month Renal EDV** | 26.34 | 10.3-73.14 | 22.415 | 12.1-37.64 | 0.156 |
| **Postop 1^st^month Renal RI** | 0.65 | 0.47-0.88 | 0.7 | 0.48-0.76 | **0.016** |

*PSV: Peak systolic velocity, EDV: End-diastolic velocity, RI: Resistive index, DJ: Double J, Postop: Postoperative.*
