# Supplementary material for: The effect of endoscopic renal and ureteral stone surgeries on renal blood flow in children: a prospective trial
Source: Urolithiasis. 2024 Jun 7;52(1):84. doi: 10.1007/s00240-024-01578-z (PMC11161530; doi:10.1007/s00240-024-01578-z)
Supplement: Supplementary file 5 — Supplementary Material 5: table 5 Comparison of preoperative, postoperative first day and month RDUS parameters of the affected kidney according to stone burden. [file 240_2024_1578_MOESM5_ESM.docx]

**Supplementary Table** **5.** Comparison of preoperative, postoperative first day and month RDUS parameters of the affected kidney according to stone burden

|  | **Single Stone**  **(n: 24)** | | **Multiple Stone**  **(n: 21)** | | **P value** |
| --- | --- | --- | --- | --- | --- |
|  | Median | Min.-Max. | Median | Min.-Max. |  |
| **Preop Segmental PSV** | 30.38 | 16.05-79.8 | 34.03 | 18-60.5 | 0.495 |
| **Preop Segmental EDV** | 11.60 | 5.07-39.16 | 9.78 | 6.11-18.8 | 0.133 |
| **Preop Segmental RI** | 0.61 | 0.38-0.74 | 0.63 | 0.53-0.75 | 0.198 |
| **Preop Renal PSV** | 86.55 | 32.4-159.6 | 89.80 | 38.4-117.27 | 0.453 |
| **Preop Renal EDV** | 29.93 | 8-67.9 | 30.84 | 11.4-43.7 | 0.982 |
| **Preop Renal RI** | 0.65 | 0.48-0.77 | 0.65 | 0.55-0.81 | 0.648 |
| **Postop 1^st^ day Segmental PSV** | 28.15 | 19.69-94.47 | 31.52 | 18-64.40 | 0.847 |
| **Postop 1^st^ day Segmental EDV** | 12.24 | 5.97-39.6 | 11.66 | 7.18-24.70 | 0.776 |
| **Postop 1^st^ day Segmental RI** | 0.60 | 0.46-0.76 | 0.62 | 0.41-0.72 | 0.829 |
| **Postop 1^st^day Renal PSV** | 66.13 | 34.6-199.81 | 77.35 | 37.6-125.60 | 0.802 |
| **Postop 1^st^day Renal EDV** | 25.25 | 11.4-64.5 | 27.10 | 11.56-47.20 | 0.682 |
| **Postop 1^st^day Renal RI** | 0.66 | 0.49-0.86 | 0.64 | 0.52-0.79 | 0.592 |
| **Postop 1^st^month Segmental PSV** | 32.07 | 16.4-98.89 | 33.56 | 22-65.17 | 0.946 |
| **Postop 1^st^month Segmental EDV** | 11.99 | 6.4-40.42 | 11.70 | 5.9-26.07 | 0.820 |
| **Postop 1^st^ month Segmental RI** | 0.61 | 0.43-0.70 | 0.64 | 0.52-0.78 | 0.350 |
| **Postop 1^st^ month Renal PSV** | 86.09 | 37.1-194.06 | 79.75 | 34.9-175.23 | 0.891 |
| **Postop 1^st^ month Renal EDV** | 26.22 | 12.90-73.14 | 25.15 | 10.3-57.93 | 0.413 |
| **Postop 1^st^ month Renal RI** | 0.62 | 0.47-0.76 | 0.68 | 0.58-0.88 | **0.026** |

*PSV: Peak systolic velocity, EDV: End-diastolic velocity, RI: Resistive index, DJ: Double J. Postop: Postoperative*
