# Supplementary material for: The effect of endoscopic renal and ureteral stone surgeries on renal blood flow in children: a prospective trial
Source: Urolithiasis. 2024 Jun 7;52(1):84. doi: 10.1007/s00240-024-01578-z (PMC11161530; doi:10.1007/s00240-024-01578-z)
Supplement: Supplementary file 6 — Supplementary Material 6: table 6 Comparison of preoperative, postoperative first day and month RDUS parameters of the affected kidney according to history of previous intervention for urinary system stones [file 240_2024_1578_MOESM6_ESM.docx]

**Supplementary Table** **6.** Comparison of preoperative, postoperative first day and month RDUS parameters of the affected kidney according to history of previous intervention for urinary system stones

|  | **Absent**  **(n: 31)** | | **Present**  **(n: 14)** | | **P value** |
| --- | --- | --- | --- | --- | --- |
|  | Median | Min.-Max. | Median | Min.-Max. |  |
| **Preop Segmental PSV** | 30.36 | 16.05-79.8 | 33.71 | 20-65.53 | 0.980 |
| **Preop Segmental EDV** | 11.20 | 5.07-25.50 | 11.27 | 6.11-39.16 | 0.759 |
| **Preop Segmental RI** | 0.62 | 0.50-0.74 | 0.62 | 0.38-0.75 | 0.676 |
| **Preop Renal PSV** | 89.80 | 32.4-159.6 | 87.50 | 38.4-117.27 | 0.873 |
| **Preop Renal EDV** | 31.26 | 8-67.9 | 27.59 | 11.4-43.7 | 0.405 |
| **Preop Renal RI** | 0.65 | 0.54-0.77 | 0.65 | 0.48-0.81 | 0.325 |
| **Postop 1^st^ day Segmental PSV** | 30.00 | 18-94.47 | 29.46 | 20.28-60.61 | 0.624 |
| **Postop 1^st^ day Segmental EDV** | 12.40 | 5.46-39.6 | 11.10 | 7.18-22.24 | 0.433 |
| **Postop 1^st^ day Segmental RI** | 0.61 | 0.46-0.76 | 0.61 | 0.50-0.74 | 0.650 |
| **Postop 1^st^day Renal PSV** | 69.93 | 34.6-199.81 | 74.67 | 36.9-109.72 | 0.816 |
| **Postop 1^st^day Renal EDV** | 27.40 | 11.4-64.5 | 25.22 | 11.56-32.58 | 0.216 |
| **Postop 1^st^day Renal RI** | 0.65 | 0.49-0.75 | 0.65 | 0.52-0.86 | 0.641 |
| **Postop 1^st^month Segmental PSV** | 34.03 | 16.4-98.89 | 31.56 | 18.80-59.33 | 0.371 |
| **Postop 1^st^month Segmental EDV** | 12.38 | 5.9-40.42 | 11.07 | 5.9-17.54 | 0.088 |
| **Postop 1^st^ month Segmental RI** | 0.61 | 0.43-0.69 | 0.64 | 0.52-0.78 | 0.137 |
| **Postop 1^st^ month Renal PSV** | 75.18 | 35.90-194.06 | 87.28 | 34.9-146.85 | 0.825 |
| **Postop 1^st^ month Renal EDV** | 26.34 | 12.10-73.14 | 24.92 | 10.3-40 | 0.391 |
| **Postop 1^st^ month Renal RI** | 0.65 | 0.47-0.73 | 0.675 | 0.57-0.88 | **0.048** |

*PSV: Peak systolic velocity, EDV: End-diastolic velocity, RI: Resistive index, DJ: Double J. Postop: Postoperative*
